# Rapid Clathrin-Mediated Uptake of Recombinant α-Gal-A to Lysosome Activates Autophagy

**DOI:** 10.3390/biom10060837

**Published:** 2020-05-30

**Authors:** Margarita M. Ivanova, Julia Dao, Neil Kasaci, Benjamin Adewale, Jacqueline Fikry, Ozlem Goker-Alpan

**Affiliations:** Lysosomal and Rare Disorders Research and Treatment Center, Fairfax, VA 22030, USA; jdao@ldrtc.org (J.D.); neilkass@gmail.com (N.K.); adewalejr.ben@gmail.com (B.A.); jfikry@ldrtc.org (J.F.); ogoker-alpan@ldrtc.org (O.G.-A.)

**Keywords:** Fabry disease, enzyme replacement therapy, alpha-galactosidase A, endocytosis, lysosome, IGF2R/M6P, clathrin, chloroquine

## Abstract

Enzyme replacement therapy (ERT) with recombinant alpha-galactosidase A (rh-α-Gal A) is the standard treatment for Fabry disease (FD). ERT has shown a significant impact on patients; however, there is still morbidity and mortality in FD, resulting in progressive cardiac, renal, and cerebrovascular pathology. The main pathway for delivery of rh-α-Gal A to lysosome is cation-independent mannose-6-phosphate receptor (CI-M6PR) endocytosis, also known as insulin-like growth factor 2 receptor (IGF2R) endocytosis. This study aims to investigate the mechanisms of uptake of rh-α-Gal-A in different cell types, with the exploration of clathrin-dependent and caveolin assisted receptor-mediated endocytosis and the dynamics of autophagy-lysosomal functions. rh-α-Gal-A uptake was evaluated in primary fibroblasts, urine originated kidney epithelial cells, and peripheral blood mononuclear cells derived from Fabry patients and healthy controls, and in cell lines HEK293, HTP1, and HUVEC. Uptake of rh-α-Gal-A was more efficient in the cells with the lowest endogenous enzyme activity. Chloroquine and monensin significantly blocked the uptake of rh-α-Gal-A, indicating that the clathrin-mediated endocytosis is involved in recombinant enzyme delivery. Alternative caveolae-mediated endocytosis coexists with clathrin-mediated endocytosis. However, clathrin-dependent endocytosis is a dominant mechanism for enzyme uptake in all cell lines. These results show that the uptake of rh-α-Gal-A occurs rapidly and activates the autophagy-lysosomal pathway.

## 1. Introduction

The past two decades have been highlighted by impressive progress in the treatment of lysosomal storage disorders (LSD) with the development of innovative therapies, including enzyme replacement therapy (ERT) [1]. The success of ERT in Gaucher disease stimulated the expansion of targeted enzyme replacement for other LSD. Currently, ERT is the first specific treatment for several LSD, including Anderson–Fabry disease (FD) [2]. FD is an X-linked disorder that results from a mutation of the gene (*GLA*) that encodes the lysosomal enzyme α-Galactosidase A (α -Gal-A).

The symptoms of FD are heterogeneous and include renal failure, cardiovascular disease, cerebrovascular complications, dermatologic manifestations, ocular and hearing complications, auditory, and neurologic complications [3,4,5]. Cardiovascular pathology and end-stage renal disease are the leading causes of death in male FD. The involvement of the central nervous system in FD increases the incidence of ischemic strokes and causes a significant decrease in lifespan in Fabry patients. The life expectancy of male patients with FD, if untreated, is approximately 40–42 years. Heterozygous females have higher residual α–Gal A activities. However, females develop clinical manifestations of varying severity and also have a reduced life span [6].

The α -Gal-A deficiency leads to the accumulation of globotriasylceramide (Gb3) in lysosomes of many cell types, including neurons, cardiomyocytes, and renal cells. ERT is effective in reducing glycolipid substrate accumulation in cells and appears to slow the progression of the FD [2,3]. Not all organs or tissues equally benefit from ERT. In general, the effectiveness of ERT becomes limited when treatment is started in adults. ERT can stabilize kidney function in patients with stage 1 or 2 chronic kidney disease; however, ERT is not effective with advanced kidney pathology, glomerular fibrosis, and sclerosis [7,8,9]. Additionally, ERT preserves the cardiac structure and heart function if treatment is initiated before the development of significant cardiac involvement. However, many patients with cellular hypertrophy in cardiomyocytes and vascular smooth muscle cells associated with tissue fibrosis still experience progressive cardio complications [10,11].

Intravenously-administrated recombinant enzyme uptakes by cells through the cell surface receptor IGF2R/M6P. It is shown that, in most ERT, the IGF2R/M6P-mediated endocytosis is crucial for efficient enzyme delivery [12,13,14]. IGF2R/M6P is a bifunctional receptor that mediates binding and endocytosis of proteins via the clathrin-associated pathway [15,16]. IGF2R/M6P is essential for several cell signaling processes, including lysosomal enzyme trafficking from trans-Golgi apparatus, clearance, activation of growth factors, endocytosis-mediated delivery of macromolecules to the lysosomes [16]. IGF2R/M6P is expressed in most tissues, with relatively higher expression in kidneys and lungs, which makes this receptor attractive for the development of intracellular drug delivery. The function of IGF2R/M6P is to bind and transport the M6P enzyme to lysosomes and has been utilized for the therapeutic applications of ERT. For FD disease, two rh-α-gal-A enzymes, agalsidase beta and agalsidase alfa, are used for ERT, and both enzymes contain M6P [17,18].

The current challenge of ERT is that treatment does not produce satisfactory results when initiated in patients with advanced stages of the disease. A better understanding of the mechanism of enzymatic uptake in different tissues and cell types is needed to improve the therapeutic outcome of ERT for FD. In this study, we compared the enzyme uptake efficiency in primary cells derived from different tissue sources—PBMC, fibroblasts, kidney epithelial cells derived from FD patients—with cell lines of different origin—HEK293, HUVEC, and HTP1 cells.

We demonstrated that uptake and transport of recombinant enzyme to lysosome is the immediate activation of autophagy. Efficiency and the maximum capacity of uptake rh-α-Gal-A is time- and cell type-specific. The FD fibroblasts demonstrated maximum enzyme uptake and HUVEC cells—the lowest enzyme uptake efficiency. IGF2R/M6P plays an essential role in the delivery of rh-α-Gal-A to the lysosome via clathrin- and, to a lesser extent, caveolae-mediated endocytosis.

## 2. Materials and Methods

### 2.1. Chemicals

Genistein, chloroquine, monensin, nocodazole (cat no. 1228) were purchased from Tocris Bioscience (Bristol, UK). The recombinant rh-α-Gal-A enzyme was from commercial source: “Fabrazyme” from Sanofi/Genzyme Corporation (Cambridge, MA, USA). Biochemical and pharmacological characteristics of commercial rh-α-Gal-A described [18]. We used rh-α-Gal-A from leftover vials after reconstitution for patient use.

### 2.2. Subjects

Primary cells derived from FD patients have been used for the study. The diagnosis of FD was confirmed by clinical presentations and enzyme, and molecular analysis. All patients gave a written informed consent form for the collection and analysis of their data. The clinical protocol was approved by the ethics committees and data protection agencies (WIBR l #20131424).

### 2.3. Cell Lines

HEK293, HUVEC, THP-1, and wild-type primary dermal fibroblast cells were purchased from American Type Tissue Collection (ATCC; Manassas, VA, USA). Primary Dermal Fibroblasts cells were grown in Media 106 with the addition of LSGS kit (S-003-10) (ThermoFisher, Rockford, IL, USA) and used between passage 4-10. HEK293 cells were maintained in 5% FBS with Improved Minimum Essential Medium (IMEM) (ThermoFisher, Rockford, IL, USA), THP-1 cells were grown in RPMI (ThermoFisher, Rockford, IL, USA) with 10% FBS following the manufacturer’s recommendation. HUVEC cells were grown in vascular cell basal medium with the addition of VEGF endothelial cell growth kit (ATCC; Manassas, VA, USA), and used between passages 3 and 8.

### 2.4. Isolation and Growth of Primary Skin Fibroblasts

Tissue samples were obtained from two patients carrying V269E and Y134D mutations in the *GLA* gene (Table S1). Skin biopsies were placed into a 50 mL conical tube and washed in PBS with 1% penicillin/streptomycin solution (ThermoFisher Scientific, Rockford, IL, USA). Skin fibroblasts were cultured as per standard methodology with complete Media 106 (Media 106, Low Serum Growth Supplement Kit and normocin, ATCC) [19]. LSGS specifically designed for the growth of dermal fibroblasts and endothelial cells. Fibroblast cells were sub-cultured at a split ratio 1:4 and used between passages 4 and 10. Cells were not immortalized.

### 2.5. Isolation, Purification, and Growth of Urine-Derived Kidney Cells

Fresh 25–50 mL of midstream urine samples were collected from two male patients with FD carrying deletion mutation c.194+1/195-1 and C2233Y mutations in the *GLA* gene and healthy controls (Table S1) The samples were processed immediately followed the protocol [19]. Briefly, urine samples were centrifuged at 400× *g* for 10 min, washed with PBS containing 1% ampicillin/streptomycin, and cell pellets were collected. Then, cells were plated in a 24-well dish with renal epithelial cell basal media supplemented with renal epithelial cell growth kit (ATCC) specifically designed for the growth of renal epithelial cells and mixed of antibiotics, normocin (InvivoGen, San Diego, CA, USA). While most cells from urine failed to attach, kidney epithelial cells attached to plate surfaces. The culture media was changed every 2–3 days until cells formed colonies. The cells were split using 0.05% Trypsin when culture cells reached the formation of large colonies. After the first passages, kidney epithelial cells (UKEC) were continuously grown in complete renal epithelial cell basal media. The cell culture subsets of composition and characteristics were analyzed. As expected, we detected a significant decrease of α-Gal A activity in patient samples compared to controls (Table S1). RT-PCR reveals the presence of epithelial markers E-cadherin (CDH1) and epithelial cell adhesion molecule (EPCAM) and the absence of podocyte markers: Podocin (NPHS2) and Nephrin (NPHS1) [20]. The maximum passage number was used 6–8 passages, or until cells were unable to reach confluence and started to undergo apoptosis. Cells were not immortalized.

### 2.6. Isolation, Purification, and Culture of Peripheral Blood Monocytes (PBMC)

PBMC were purified from blood samples from patients with Fabry disease using Lymphoprep™ reagent and SepMate™ tubes (Stemcell Technologies, Vancouver, BC, Canada) following the manufacturer’s protocol. Lymphoprep™ was added to the lower compartment of the SepMate tube. Blood was mixed with PBS + 2% FBS in a 1:1 ratio, then layered on top of Lymphoprep™ following the company protocol. Samples were centrifuged at 800× *g* for 20 min at 18 °C with the brake off. The upper plasma layer was discarded. The PBMCs layer was removed carefully, then washed with PBS and centrifuged at 300× *g* for 8 min at room temperature between each wash. Isolated PBMC were treated in 5% CO_2_ in phenol red-free RPMI media with 10% FBS. PBMC always was used fresh following the experiments.

### 2.7. Treatment of Cells with rh-α-Gal-A and Other Chemicals

The cells were split, and cultures using the recommended media for specific cell lines were established 24 h before the treatments. DMSO was used as the vehicle control for experiments with inhibitors. Cells were treated with various concentrations of rh-α-Gal-A enzymes, as shownd in the figures. For the indicated experiments, cells were pretreated with 50 µg/mL nocodazole, 200 µM chloroquine, 50 ng monensin for 30 min, and 100 µM genistein for 1 h before rh-α-Gal-A treatment.

### 2.8. Uptake rh-α-Gal-A via an Alexa Fluor^TM^ Protein Labeling Kit

Rh-α-Gal-A protein conjugates containing the Alexa Fluor dyes (488 or 555) were prepared following the manufacture’s protocol (ThermoFisher, Rockford, IL, USA). We titrated the range of enzyme activity pre- and post-labeling towards the artificial substrate 4-MUI in different volumes (Figure S1A). The log IC50 = −4.6 for unlabeled enzyme and log IC50 = −3.4 for labeled enzyme indicates that approximately 76% of the enzyme was recovered. Intracellular uptake of Alexa-Fluor-α-Gal-A was further verified qualitatively by a confocal microscope (Figure S1B). Fluorescence clusters were confirmed in fibroblast cells cultured with Alexa-Fluor-α-Gal-A conjugates and the absence of a fluorescence signal in cells cultured with free dye. Validation of uptake was investigating using co-incubating live cells with Alexa-Fluor-α-Gal-A conjugates for 1 and 3 h. LysoTracker or autophagy dyes were added 30 min prior to stop the reaction. Then cells were washed with PBS three times. Cells were visualized by fluorescent microscopy, where co-localization of green-(488)-labeled α-Gal-A protein with red-labeled lysosomes appears yellow in color-merged images. Green-labeled autophagy vesicles were co-stained with red-(550)-labeled α-Gal-A protein.

### 2.9. α-Galactosidase A Activity Assay

Cells were washed with cold PBS three times and lysed in cold H_2_O after the treatments as described above. Protein concentration was determined by the Pierce BCA protein assay kit (ThermoFisher, Rockford, IL, USA) according to the manufacturer’s manual. An activity was fluorometrically determined by incubating 10 μg/mL of samples with 5 mM 4-Methylumbelliferyl α-D-galactopyranoside and in 0.06 M phosphate citrate buffer (pH 4.7) for 1 h (Santa Cruz Biotechnology, Dallas, TX, USA). Enzyme activity was measured as described previously and is expressed as the nmol 4-MU/mg protein/time incubation or as a relative level to control, untreated samples [21,22].

### 2.10. Protein Isolation and Western Blot Analysis

Antibodies were purchased as follows: IGF-II Receptor/CI-M6PR (D3V8C) and β-actin (# 8H10D10) (Cell Signaling Technology, Danvers, MA, USA), α-Gal-A (#GTX101178) (GeneTex, Irvine, CA, USA). Whole-cell extracts (WCEs) were prepared in radioimmunoprecipitation (RIPA) buffer. Protein concentrations were determined using the BCA Protein Assay Kit (ThermoFisher, Rockford, IL, USA). Thirty micrograms (30 μg) of WCE were separated on mini protein TGX stain-free gel (Bio-Rad, Hercules, CA, USA) and electroblotted using the Trans-Blot^®^ Turbo™ Midi PVDF Transfer Packs (Bio-Rad, Hercules, CA, USA). Membranes were diluted with antibodies (1:1000 dilutions) in 5% BSA, 1 × TBS, 0.1% Tween20, and gently shaking overnight at +4 °C. The ChemiDocTM MP Imaging system (Bio-Rad) was used to visualize and quantitate optical density (IOD). The IODs of bands of interest were normalized to the loading control actin used in the same blot [8], and the normalized value of the controls was set to 1 for a comparison between separate experiments.

### 2.11. Autophagy Assay

The DALgreen Autophagy detection kit (Dojindo Laboratories, Kumamoto, Japan) was used according to the manufacture’s protocol to quantify autophagic vesicle formation and Hoechst 33342 dye as an index of the nucleus. The resulting fluorescence was visualized by fluorescent microscopy (Evos^R^ digital microscope, Evos, Hatfield, PA, USA).

### 2.12. Measurement of Lysosome Levels

The LysoTracker Red (LifeTechnology, ThermoFisher, Rockford, IL, USA) assay was used as briefly described. LysoTracker (50 nM) was added to the cells as a fluorescent acidophilic probe for the labeling of the acidic organelles. After 30 min staining, cells were stained with Hoechst, and washed three times with PBS. The resulting fluorescence was visualized by fluorescent microscopy (Evos^R^ Digital microscope, Evos, Hatfield, PA, USA).

### 2.13. RNA Isolation and Quantitative Real-Time-PCR (qPCR)

RNA was extracted from cells using the Quick-RNA kit (Zymo Research, Irvine, CA, USA). The Luna^®^ Universal Probe One-Step RT-qPCR Kit was used to reverse-transcribe RNA using random hexamers primers. Individual samples were run in triplicate, and mRNA levels were compared to the loading control, GADPH, using StepOnePlus™ Real-time PCR System (ThermoFisher Scientific, Rockford, IL, USA). The E-cadherin primers [23], EpCAM [24], and two pairs of IGF2R/M6P [24,25] primers were purchased from Eurofins Genomics. Analyses and fold differences were determined using the comparative CT method. Fold change was calculated from the ΔΔCT values with the formula 2^−ΔΔCT^ relative to mRNA expression in the untreated control.

### 2.14. Immunofluorescence Microscopy Analysis

Scatter plots, Person’s correlation coefficient, and colocalization threshold were obtained using ImageJ-win64 plug-in intensity correlation analysis. The image and statistical analysis of colocalization was performed with Coloc 2 Fiji’s plugin and colocalization threshold (Figure S3). In a scatter plot, the intensity distribution of the two channels are plotted (X vs. Y) and a diagonal line indicates proportional co-distribution, where R^2^ = 1 is a perfect positive linear relationship between two fluorescence intensities.

### 2.15. Interactive 3D Surface Plots Analysis

ImageJ plugins (NIH, Bethesda, MD, USA) with the option of 3D surface plot techniques of image data was used to analyze the intensity projection (Z coordinates) of autophagy staining. Pixels with higher intensity values lay higher on the Z axis. Parameters of the surface are plotted as 100% of the polygon multiplier, drawn in wireframe, shaded, and drawn on the axis.

## 3. Results

### 3.1. The Efficiency of Enzyme Uptake Is Cell Type-Specific

**Figure 1:**
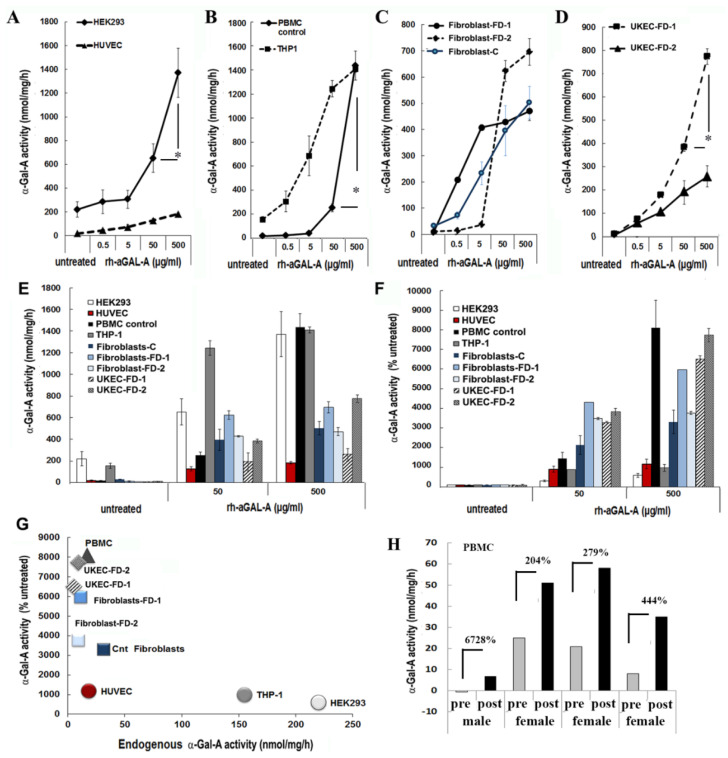
The efficiency of the delivery of recombinant α-Gal A enzyme (rh-α-Gal A). (**A**) HEK293 and HUVEC, (**B**) THP1 and control PBMC, (**C**) fibroblasts, and **(D**) UKEC cells were treated with increasing concentration of rh-α-Gal A for 1 h. A total α-Gal A enzyme activity level was determined using 4-MU. * *p < 0.05* 50 vs. 500 µg/mL of α-Gal A treatment (**E**,**D**) Comparing the total absolute (**E**) and relative (**D**) enzyme activity levels. Measurements performed at the concentrations of 50 and 500 µg/mL were compared with the enzyme activity in untreated samples. (**G**) Correlation analysis between endogenous enzyme concentration and enzyme uptake efficiency. (**H**) α-Gal A activity was measured immediately following ERT treatment. Blood was collected before (pre) and after (post) ERT infusion and PBMCs were isolated from FD patients, one male, and three females. Values represent 4-MU pre- and post-infusion levels. Samples were measured in triplicate.

To test the hypothesis that the efficiency of rh-α-Gal-A uptake is cell type-specific, seven cell lines of different origins were examined (Table S1). HEK293, primary fibroblasts derived from healthy controls, and FD patients were selected because these cells are prevalent for studies of molecular mechanisms of drug delivery, including FD [26]. Since FD notably affects the vascular endothelium [27], human umbilical vein endothelial cells (HUVEC) were selected as a vascular model to study the delivery of the recombinant enzyme. Monocyte cell lines derived from a patient with acute monocytic leukemia (THP-1), PBMC derived from control, and FD patients were selected as models of the hematopoietic system. Since FD affects the kidney in almost all males and many females, the kidney epithelial cells isolated from urine were selected to study the mechanism of enzyme uptake. Cell lines and primary cells were treated for 1 h using a range of rh-α-Gal-A enzyme: from 0.05 g/mL to 500 g/mL (Figure 1). It appears that the efficiency of recombinant enzyme uptake was cell-type dependent (Figure 1A–C). In UKEC derived from two FD patients, the maximum uptake capacity differed greatly between patients with different *GLA* genotypes. The robust, but different, amplitude of the dose-dependent response was demonstrated in HEK293, PBMC, THP-1, fibroblasts, and UKEC cells. The rh-α-Gal-A enzyme uptake was less efficient in HUVEC cells (Figure 1A). Under this condition, our results demonstrated that enzyme activity reached a plateau at a concentration of 50 ug/mL in HUVEC, THP-1, fibroblasts, and UKEC-FD-2 cells (Figure 1C). The highest level of α-Gal-A activity approximated 1200–1600 nmol/mg/h and was observed in HEK293, control PBMC, and THP-1 cells. Medium level in the range of 330–800 nmol/mg/h of α-Gal-A activity was observed in primary fibroblasts and UKEC (Figure 1E). The lowest level was observed in HUVEC cells. Based on the fact that different cells have the different activity of endogenous α-Gal-A, the relative enzyme uptake was calculated as the percentage of treated vs. untreated cells for each cell line (Figure 1F). PBMC, FD derived fibroblasts, and UKEC cells showed greater than 3000-fold increase in α-Gal-A activity level compared with untreated cells (Figure 1F). Since the uptake of rh-α-Gal-A less efficient in cells with high endogenous α-Gal-A activity, we analyzed the correlation between endogenous enzyme activity and enzyme uptake efficiency. Uptake of rh-α-Gal-A was higher in cells with the lowest endogenous enzyme activity. Conversely, cells with high endogenous α-Gal-A activity demonstrated less effective uptake of the recombinant enzyme (Figure 1G). Moreover, HUVEC cells showed the lowest response to rh-α-Gal-A treatment (Figure 1A,D,G).

Next, we assessed ERT efficiency in the in vivo model. Immediately before and after ERT infusion, blood was collected from four FD patients (one male and three females), and α -Gal-A activity was measured. Plasma rh-α-Gal-A activity after ERT infusion was very similar among the four subjects and averaged 4502 ± 404 nmol/mL/h for all samples. In contrast, enzyme uptake efficiency into PBMC differed significantly among patients. The highest percentages of enzyme uptake were observed in PBMC derived from FD male with zero enzyme activity and female with the lowest endogenous α-Gal-A activity (Figure 1H). However, the highest total α-Gal-A activity was observed in cells from patients with the highest endogenous enzyme activity (Figure 1H).

### 3.2. The Uptake of rh-α-Gal-A Occurs On a Minute to Hours’ Time Scale

**Figure 2:**
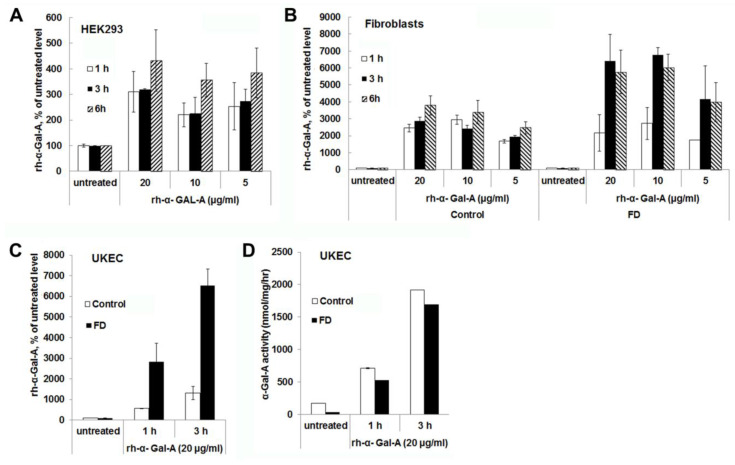
The rapid uptake of rh-α-Gal A. HEK293 (**A**), fibroblasts (**B**), and UKEC (**C**,**D**) cells were treated with the indicated concentration of rh-α-Gal A in a time-dependent manner. The enzyme assay was performed to determine the relative α-Gal A enzyme level. FD fibroblasts and UKEC cells represent data from FD-1 and FD-2 cell lines respectably. Values are average ±STDEV of minimum three experiments. * *p* < 0.01 vs. untreated control.

Significant increases in α-Gal-A activity were detected within 1 h of treatment with the recombinant enzyme in all cell lines, including cells derived from FD patients. After 1 h, the α-Gal-A activity did not change drastically within 3h of treatment in HEK293 and control fibroblasts; however, the maximum uptake was observed within 6 h of treatment (Figure 2A,B). FD fibroblasts showed the maximum level of rh-α-Gal-A activity with 3 h treatment (Figure 2B,C). Significant increases in α-Gal-A activity were observed in control, and FD derived UKEC after 1h treatment with increased uptake after 3 h treatment (Figure 2C). Interestingly, both control and FD UKEC cells showed similar enzyme activity of α-Gal-A after 1 h and 3 h treatment with the recombinant enzyme (Figure 2D). Thus, this result indicates that the maximum α-Gal-A capacity for control and FD UKEC are similar.

### 3.3. IGF2R/M6P Increase in HEK293 After Six Hours of Enzyme Uptake

**Figure 3:**
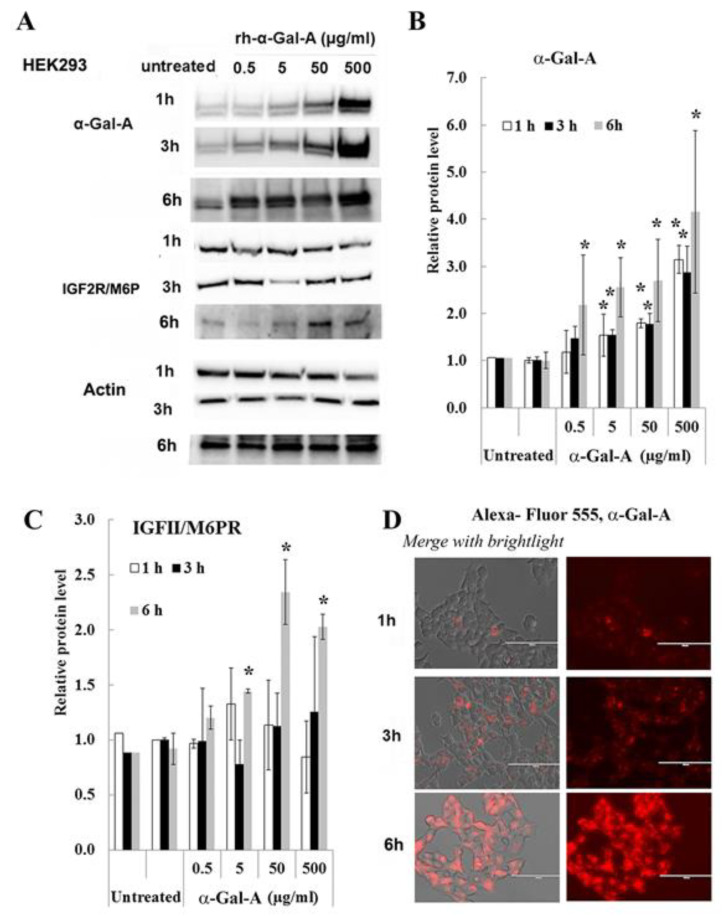
IGF2R/M6P decreases during rapid uptake of rh-α-Gal A. HEK293 cells were treated with the indicated concentrations of rh-α-Gal A for 1, 3, and 6 h. (**A**) Western blot (30 µg WCE) shows that uptake of rh-α-Gal A protein increases in concentration- and time-dependent manners. IGF2R/M6P increased after 6 h treatment. Membranes were reprobed for actin for normalization. (**B**) Quantitation of the relative level of α-Gal A. (**C**) Quantitation of relative level IGF2R/M6P. (**D**)) Immunofluorescence images of time course treated HEK293 cells with Alexa-Fluor-α-Gal-A conjugates. Bars: 100 μm.

Effectiveness of ERT with rh-α-Gal-A is dependent on recognition of mannose 6-phosphate (M6P) residues on the enzyme by the widely distributed IGF2R/M6P receptor [13,17]. The half-life of IGF2R/M6P is approximately *t*_½_ ∼ 20 h, while the receptor cycles between trans-Golgi network, endosomes and the plasma membrane where it loads and unloads ligands [28]. The molecular mechanism of the cycle includes packaged enzymes into lysosomes, whereas the “free” IGF2R returns to the Golgi apparatus or move to the plasma membrane. To test the IGF2R/M6P cycling during uptake of rh-α-Gal-A, HEK293 cells were treated for 1, 3, and 6 h with increasing concentration of rh-α-Gal-A (Figure 3). The highest rh-α-Gal-A uptake was detected after 6h treatment without toxicity effect (Figure 3A,B, Figure S1C). Treatment HEK293 cells with Alexa-Fluor-α-Gal-A conjugates confirmed the highest rh-α-Gal-A uptake after 6h treatment (Figure 3D). Interestingly, the increasing level of IGF2R/M6P protein was detected after 6h treatment (Figure 3C). Accordingly, 6 h treatment with an increasing concentration of rh-α-Gal-A increased IGF2R/M6P expression of mRNA (Figure S2).

### 3.4. Transport of rh-α-Gal-A Is Achieved by Clathrin and Caveolae-Mediated Endocytosis in a Cell Type-Specific Manner

**Figure 4:**
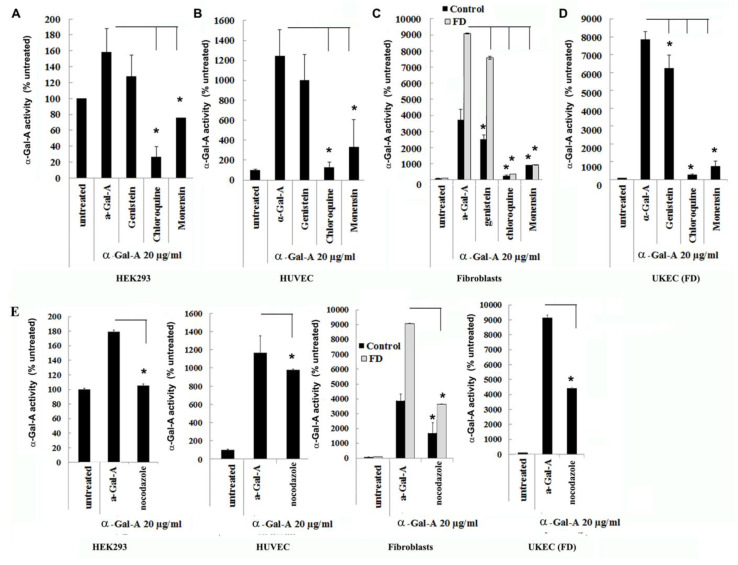
The rapid uptake of rh-α-Gal-A is achieved by clathrin and caveolae-mediated endocytosis. HEK293 (**A**), HUVEC (**B**), fibroblasts (**C**), and UKEC (**D**) cells were co-treated with the indicated concentration of rh-α-Gal A and inhibitors genistein, chloroquine, and monensin for 1 h. The enzyme assay was performed to determine the relative α-Gal A enzyme level. (**E**) HEK293, HUVEC, fibroblast, and UKEC cells were treated with the microtubule inhibitor, nocodazole. The enzyme assay was performed to determine the relative α-Gal A enzyme level. Values are average ±STDEV of minimum three experiments. * *p* < 0.01 vs. untreated control.

**Figure 5:**
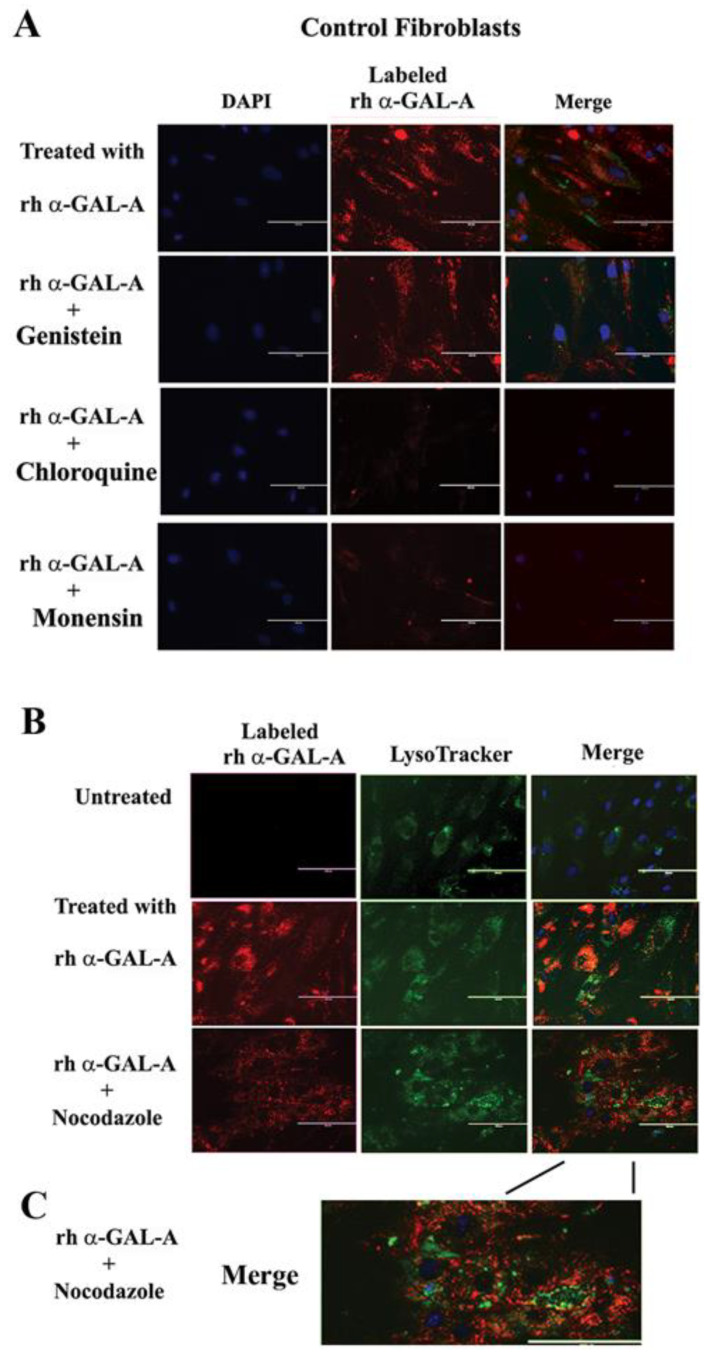
The trafficking of the recombinant enzyme is inhibited in the presence of chloroquine, monensin, and nocodazole. Control fibroblasts were co-treated with fluorescence-labeled rh-α-Gal-A and inhibitors genistein, chloroquine, monensin, and nocodazole. (**A**) Immunofluorescence images of control fibroblasts treated with fluorescence-labeled rh-α-Gal-A (red). Bars: 200 μm. (**B**) Immunofluorescence images of control fibroblasts treated with fluorescence-labeled rh-α-Gal-A (red) and stained with LysoTracker (green). Bars: 200 μm. (**C**). The magnified merge image of cells with Alexa-Fluor-α-Gal-A (red) and LysoTracker (green) after nocodazole treatment.

Endocytosis, followed by lysosomal transport, support numerous cellular functions and has been used for intracellular delivery of recombinant enzymes. For the majority of cells, clathrin-coated and caveolae-mediated endocytosis are the most common uptake mechanisms [29]. However, the contribution of clathrin and caveolar pathways to endocytosis has been shown to differ between cell types and tissues, and that can contribute to the failure to efficiently deliver the recombinant enzyme to some organs [30]. We evaluated potential changes in the trafficking of the recombinant enzyme via clathrin and caveolae endocytosis. For this purpose, clathrin inhibitors (chloroquine and monensin) and caveolae inhibitor (genistein) have been used. HEK293, HUVEC, primary fibroblasts, and UKEC cells were pretreated with 100 µM of genistein, 100 µM of chloroquine, and 50 µM of monensin for 1h. The following pretreatment cells were incubated with 20 µg/mL rh-α-Gal-A plus inhibitors for 3h. Chloroquine and monensin blocked uptake of recombinant enzyme, indicating that the clathrin-mediated endocytosis is involved in recombinant enzyme delivery (Figure 4A–D and Figure 5A). Genistein (caveolae inhibitor) partially suppressed transport of rh-α-Gal-A in fibroblasts and UKEC cells, indicating that the caveolae-mediated endocytosis is partially involved in enzyme delivery (Figure 4A–D and Figure 5A).

### 3.5. Activator of Microtubule Depolymerization, Nocodazole, Blocked rh-α-Gal-A Uptake

Since microtubule-based active transport is crucial for trafficking clathrin-coated vesicles and plays a role in the relocation and distribution of lysosomes [31], it should play an essential role in the transport of recombinant enzyme to the lysosomes. To investigate the role of microtubules in the transport of recombinant enzyme, we depolymerized the microtubule cytoskeleton by treating cells with 50 ng/mL of nocodazole. After 30 min of pretreatment, cells were treated with 20 µg/mL rh-α-Gal-A. Nocodazole inhibits the increase in α-Gal-A activity after 3h treatment with the recombinant enzyme, indicating that the microtubule cytoskeleton plays a vital role in the enzyme uptake (Figure 4E). Additionally, intracellular trafficking of Alexa Fluor tagged rh-α-Gal-A (red) to lysosomes in the presence of nocodazole was examined by immunofluorescence microscopy. The colocalization between rh-α-Gal-A and lysosomes was analyzed using Coloc 2 Fiji’s plugin and colocalization threshold (ImageJ-win64) methods. Coloc 2 plugin analysis showed a reduction of that α-Gal-A colocalization with the lysosome marker after nocadozole treatment, with Pearson’s coefficient of 0.62 (untreated cells) and 0.47 (nocodazole treated cells) (Figure S3). Colocalization threshold analysis showed decreasing areas of colocalization α-Gal-A with the lysosomes in presence of nocodazole (Figure S3). Analysis of the lysosomal staining (LysoTracker, green) merged with rh-α-Gal-A (red) demonstrated that nocodazole partially inhibited the transport of the recombinant enzyme to the lysosomal compartment (Figure 5B,C, Figure S3).

### 3.6. Robust Uptake of rh-α-Gal-A to the Lysosomes Increases Autophagy

**Figure 6:**
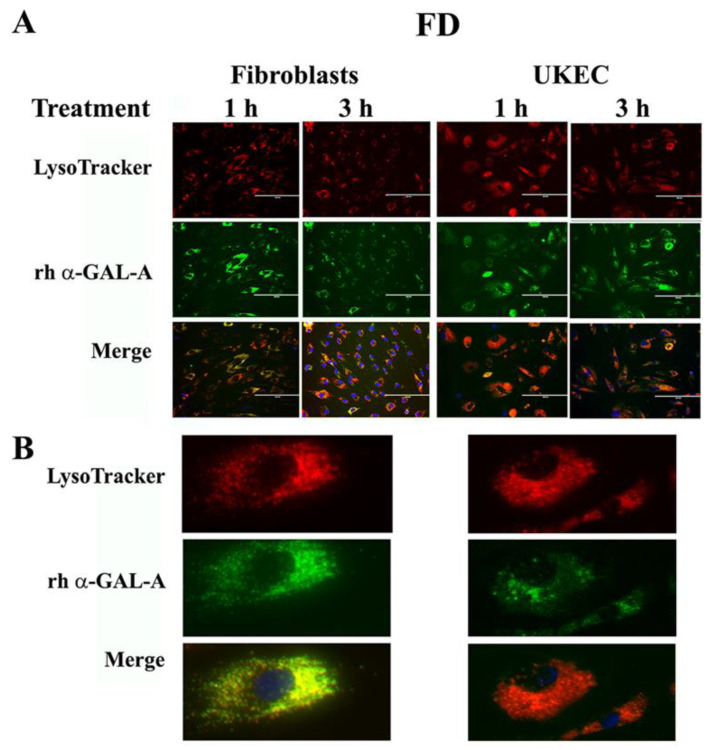
Immediate transport of rh-α-Gal-A to the lysosome. (**A**) Immunofluorescence images of FD fibroblasts and UKEC cells treated with fluorescence-labeled rh-α-Gal-A (green) and stained with LysoTracker (red). Bars: 200 μm. (**B**) Magnified image of cells with 1 h Alexa-Fluor-α-Gal-A conjugate treatment.

The final destination of rh-α-Gal-*A* is the lysosomes, where enzyme catalyzes the removal of terminal α-galactose residues. Intracellular trafficking of rh-α-Gal-A was examined by fluorescence microscopy to assess co-localization of the recombinant enzyme with lysosomes. Fibroblasts and UKEC derived from FD patients were treated with rh-α-Gal-A labeled with the Alexa Fluor dyes (488) (Figure 6A). After treatment, live cells were stained with LysoTracker and visualized under the microscope. Co-localization of Fluor-488-α-Gal-A (green color) and LysoTracker appears as the yellow-orange color after the merging of images (Figure 6A). The distinct yellow-orange color of lysosomes demonstrates that rh-α-Gal-A traffics to lysosomes in a relatively short period of time in both cell lines. Figure 6B shows an enlarged image of 1-h treated fibroblasts and UKEC cells incubated with a combination of labeled Fluor-488-α-Gal-A and LysoTracker.

**Figure 7:**
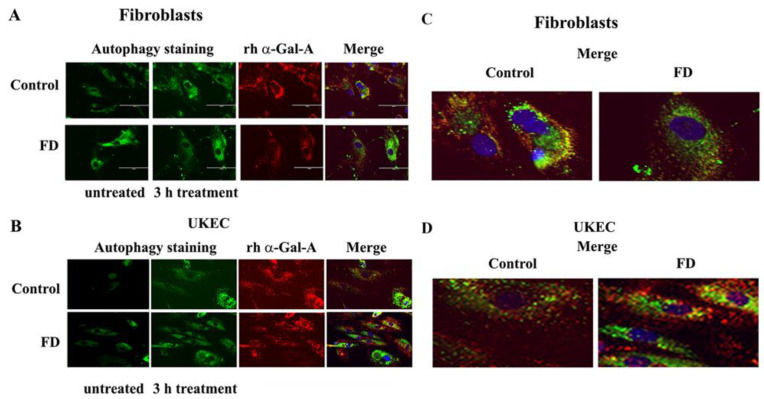
rh-α-Gal-A enhanced autolysosomal compartment. (**A**) Immunofluorescence images of control and FD fibroblasts treated with fluorescence-labeled rh-α-Gal-A (red) and stained with autophagy dye (DALgreen, green). Bar: 100 μm. (**B**). Representative images of control and FD UKEC treated with fluorescence-labeled rh-α-Gal-A (red) and stained with DALgreen (green). (**C**, **D**). Merge images of control and FD fibroblasts (**C**) and UKEC cells (**D**) treated with fluorescence-labeled rh-α-Gal-A (red) and stained with autophagy dye (DALgreen, green).

**Figure 8:**
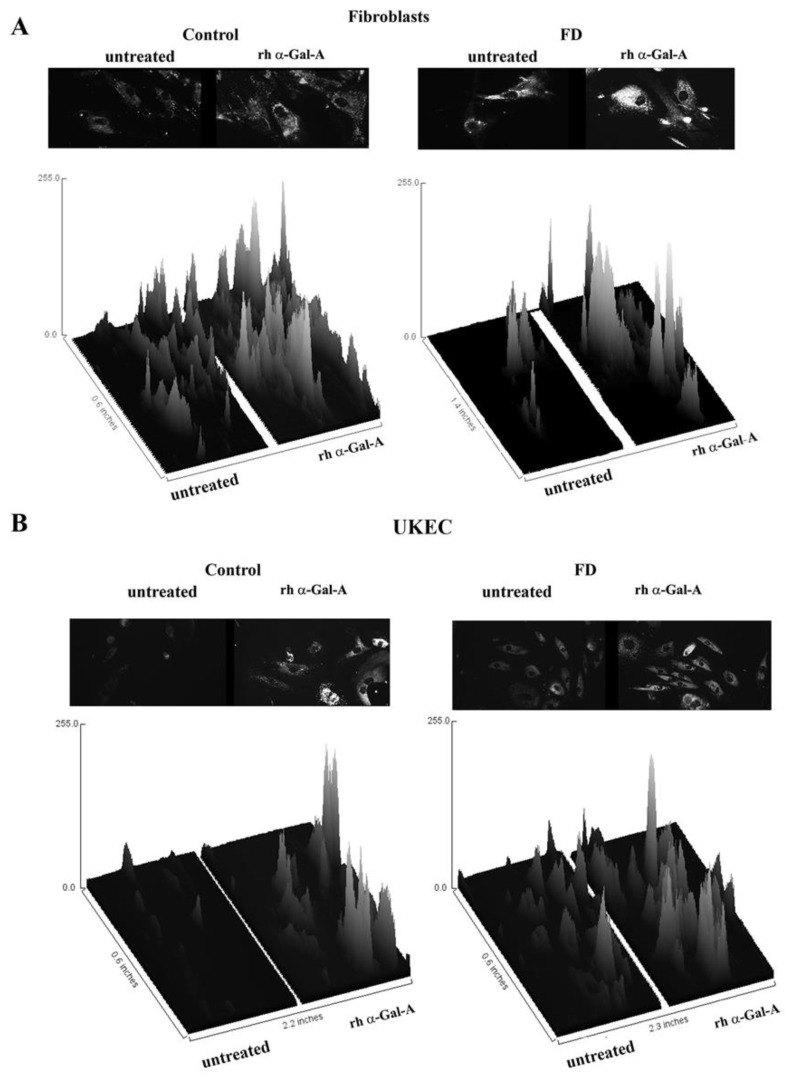
The interactive 3D surface plots displayed a three-dimensional graph of the intensities of pixels (Z) of autophagy in grayscale images. Immunofluorescence images of control vs. FD fibroblasts (**A**) and UKEC cells (**B**) treated with fluorescence-labeled rh-α-Gal-A were stained with DALgreen autophagy dye. The autophagy images used in Figure 7 were converted to 2D gray color images, and then interactive 3D surface plots were built using the ImageJ program. 2D and 3D images of control and FD fibroblast are displayed.

The disruption of the autophagy has been documented in fibroblasts, podocytes, and in PBMC derived from FD patients [20,32,33,34]. Next, we investigated the effect of rh-α-Gal-A on autophagy activation (Figure 7 and Figure 8). Fibroblasts and UKEC cells from healthy controls and FD patients were treated with rh-α-Gal-A labeled with the Alexa Fluor dye (550) (Figure 7). After 3 h treatment, live cells were stained with a DALGreen autophagy detection kit to visualize autolysosomes, a unique acidic compartment in autophagy [35]. The level of autolysosomes was increased after 3 h treatment with Alexa-Fluor-α-Gal-A conjugates in all cells (Figure 7). The interactive surface plot analysis visualized DALGreen autophagy stain configuration in 3D format. The detailed observation of maximum intensity projection (Z coordinates) showed increasing levels of autolysosomes in α-Gal-A treated cells (Figure 8). Merge analysis of autophagy staining (green) with rh-α-Gal-A (red) demonstrated that the recombinant enzyme partially co-localizes with the autolysosomal compartment (Figure 7).

## 4. Discussion

ERT has been shown to be less effective in patients who have initiated treatment at a later age and/or with advanced renal, cardiovascular, and cerebrovascular involvement. The continued accumulation of Lyso-Gb3 in the vascular endothelium and smooth muscle cells was suggested to contribute to renal failure and strokes despite ERT [36]. The mechanisms of enzyme delivery in vascular or renal cells were not fully understood. Studies of enzyme delivery or small molecules therapy in FD mostly relied on in vitro observations using HEK293 cells and fibroblasts with wild-type GLA [13,26,37]. However, molecular mechanisms of enzyme delivery to lysosomes may vary depending on the type/origin of cells.

In the present study, we show that the efficient delivery of rh-α-Gal-A is cell-type specific. Moreover, the efficiency of rh-α-Gal-A uptake is determined by endogenous enzyme activity in cells. Treated HEK293, PBMC, and THP1 cells demonstrated the highest level of α-Gal-A enzyme activity (combined endogenous and recombinant enzyme activity) without a visual toxicity effect. The calculation of uptake efficiency in different cells showed that HEK293 cells have the lowest uptake due to high concentrations of endogenous α-Gal-A. Opposite to HEK293 cells, cells with low endogenous enzyme activity, including cells derived from FD patients, showed the most efficient uptake of the recombinant enzyme. A negative correlation between the level of endogenous α-Gal-A enzyme activity and the uptake efficiency indicates that the enzyme becomes saturated in cells and that different types of cells have a different maximum capacity for α-Gal-A uptake. The only vascular type of cells, HUVEC, did not show the same trend; these cells demonstrated the lowest enzyme uptake efficiency in the presence of low endogenous α-Gal-A activity. Our study indicates that the transport of recombinant enzyme was rapid in all cell lines leading to a significant increase in α-Gal-A activity. That transport reached the maximum capacity after 1-h treatment in HEK293 and control fibroblasts. There is continued enzyme uptake thereafter in FD fibroblasts, and UKEC cells. The time course shows that the dynamics of α-Gal-A uptake are cell type-specific.

**Figure 9:**
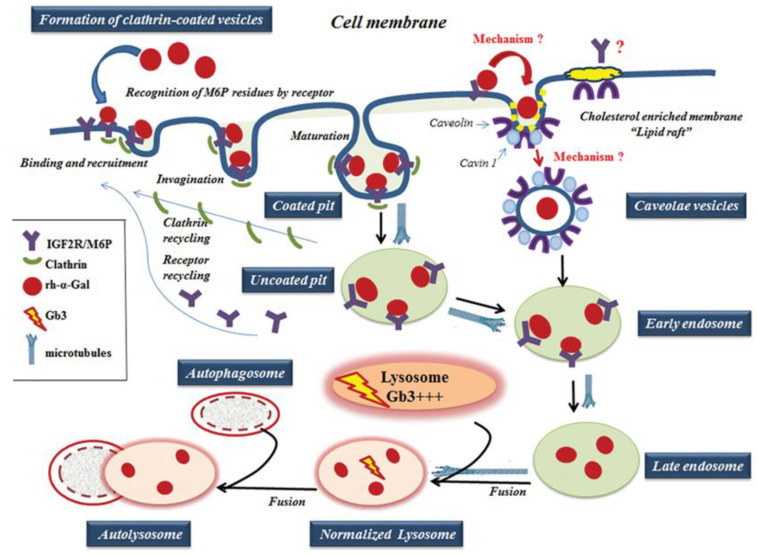
A working model of rh-α-Gal A uptake through clathrin- and caveolae-mediated endocytosis. The rh-α-Gal A contains terminal mannose residues for which conferring the high affinity for IGF2R/M6P on cells. The model proposes that the IGF2R/M6P internalizes the majority of the rh-α-Gal A through clathrin-meditated endocytosis. A small portion of IGF2R/M6P—rh-α-Gal A complex can also be uptake by caveolae-endocytic mechanisms. The microtubule cytoskeleton is involved in rh-α-Gal A endocytosis and transport enzyme to lysosomes. Early endosomes are containing recombinant enzyme mature into late endosomes, while IGF2R/M6P is recycling back to the cellular membrane. Late endosomal fuses with lysosome and delivers the therapeutic enzyme to the lysosome, which subsequently normalized Gb3 level. Normalized lysosomes fused with autophagosomes and form autolysosomes.

For ERT to be successful, the proteins in different tissues must be correctly targeted to the lysosome. The IGF2R/M6P was selected for ERT because IGF2R/M6P mediated endocytosis transports M6P-bearing recombinant enzyme to lysosomes [12]. The effective transport of recombinant α-Gal-A depends on the variation in glycosylation of M6P residues, recognized by the IGF2R/M6P receptor located on the cytoplasmic membrane (Figure 9) [18,38]. The activation of IGF2R/M6P-mediated endocytosis is the key factor responsible for efficient enzyme uptake, which also depends on the distribution of the IGF2R/M6P receptor in different cells/tissues, and the mechanism of the shuttling of the receptor between cellular membrane, Golgi complex, and lysosomes in different cells/tissues.

IGF2R/M6P receptor is localized mostly in the Golgi and endosomal compartments with less than 10% on the plasma membrane. The receptor always shuttles between intracellular compartments and cytoplasmic membrane during endocytosis (Figure 9) [14,16]. The IGF2R/M6P mediated endocytosis has been very well characterized; however, the mechanisms of IGF2R/M6P shuttle between membrane-Golgi-lysosome during rapid delivery of rh-α-Gal A to lysosomes, is unknown. HEK293 cells were used to test IGF2R/M6P cycling during rh-α-Gal-A uptake. The data verified that rapid uptake of rh-α-Gal-A is associated with increasing IGF2R/M6P level after 6 h treatment.

Although it is clear that IGF2R/M6P receptor-mediated endocytosis plays a key role in the delivery of the recombinant enzyme to the cells, the mechanisms mediating the endocytosis remain undefined and are likely to be multi-faceted. For example, in the human podocyte cell line, three endocytic receptors, IGF2R/M6P, megalin, and sortilin are responsible for the α-Gal A uptake [13]. The design of the delivery of the recombinant enzyme relies on the nature of the IGF2R/M6P membrane receptor; however, the whole complex of endocytosis machinery is involved in this process. Normally, secreted pro-enzymes are taken up by the IGF2R/M6P receptor, formed pro-enzyme/ IGF2R/M6P complexes are then internalized through clathrin-mediated endocytosis (Figure 9) [39]. Monensin and chloroquine have been used to inhibit the initial step of clathrin-dependent endocytosis: formation of the clathrin-coated pit to the clathrin-coated vesicles [40]. We showed that inhibition of initiation of clathrin-coated vesicles resulted in a significant blockade of the transport of recombinant enzyme to HEK293, HUVEC, fibroblasts, and UKEC cells. This result highlights the universal role of clathrin in the delivery of recombinant enzyme through IGF2R/M6P receptor-mediated endocytosis.

Does clathrin-dependent endocytosis provide the optimal mechanism for enzyme delivery in the FD cells? The phenomenon of decreased uptake through IGF2R/M6P endocytosis has been documented for several lysosomal storage disorders, such as in fibroblasts from Pompe and Niemann Pick patients [41,42,43]. A fluid-phase uptake study demonstrated reduced dextran uptake in Gaucher and FD fibroblasts due to the alteration of clathrin-mediated endocytosis [44]. Deficits in neurotransmitter recycling via clathrin-coated pits have been shown in mouse models of Gaucher and Batten disease [45]. Clathrin is a part of the trafficking pathway of lysosomal enzymes, and lysosomal abnormalities could be a contributing factor for inhibition of clathrin-mediated endocytosis.

Caveolar endocytosis is suggested not to be involved in endogenous lysosomal enzyme trafficking; therefore, it is less likely to be affected by lysosomal alterations [46]. The caveolae is a lipid raft that contains a high level of caveolin proteins [47]. The lipid rafts stabilize the membrane structure and contain not only lipids, but also various signaling proteins, and growth factor receptors [48]. For example, clathrin- and caveolae-dependent endocytosis controls IGF1R endocytosis [49]. We hypothesized that IGF2R/M6P could be set off via a clathrin and/or caveolae related mechanism. (Figure 9). In our study, we have shown that an alternative IGF2R/M6P-caveolar mediated endocytosis coexist with clathrin-mediated endocytosis. However, clathrin-dependent endocytosis is a dominant mechanism for enzyme uptake in all cell lines including cell lines derived from FD patients.

Lysosomes are the final destination of the recombinant enzyme. We confirmed that the trafficking of the recombinant enzyme to the lysosome is a rapid process, with significant accumulation of rh-α-Gal A within the lysosomes after 1-h treatment. Formation of the clathrin-coated vesicles and the fusion of vesicles with the lysosomes is coordinated by actin cytoskeleton and microtubules. The microtubule-depolymerizing agent, nocodazole, blocked the transport of recombinant enzyme to the lysosome. Dysfunctional lysosomes due to Gb3 accumulation can impair the trafficking of the recombinant enzyme to the lysosomes, and initiate a cascade of events that lead to autophagy abnormality in Fabry disease [32,34]. In this study, we observed that the delivery of wild type α-Gal A enzyme immediately induces the activation of autophagy in fibroblasts and UKEC cells derived from FD patients.

## 5. Conclusions

The rapid uptake and delivery of rh-α-Gal-A to the lysosome via clathrin- and, to a lesser extent, caveolae-mediated endocytosis activates autophagy in Fabry disease.

## Supplementary Materials

The following are available online at https://www.mdpi.com/2218-273X/10/6/837/s1, Figure S1: (A) The titration of rh-α-Gal-A enzyme activity pre- and post-labeling towards the artificial substrate 4-MUI. (B) Immunofluorescence images of fibroblasts treated with Alexa-Fluor 488- fluorescent dye alone and 488-fluorescence-labeled rh-α-Gal-A (green) for 1h and stained with LysoTracker (red). (C) Effects of rh-α-Gal-A on cell viability. HEK293 cells were treated with different concentrations of unlabeled rh-α-Gal-A fro 6h. The CCK-8 assay was performed to measure cell viability; Figure S2: HEK293 cells were treated 6 h with the indicated concentrations of rh-α-Gal-A, or vehicle control (untreated), and real-time-PCR analysis of IGFII/M6PR was performed: three separate experiments; Figure S3: Colocalization analysis of immunofluorescence images (Figure 5B) of control fibroblasts treated with fluorescence-labeled rh-α-Gal-A (red) and stained with LysoTracker (green) with and without nocodazole treatment. (A) Immunofluorescence images of untreated fibroblasts: fluorescence-labeled rh-α-Gal-A (left top panel), LysoTracker (left bottom panel), merge red and green images using ImageJ-win64 (right top panel) and image of co-localization areas (yellow) (right bottom panel). (B) 2D intensity histogram of the red and green pixels in the images labeled rh-α-Gal-A (channel 1, red) and LysoTracker staining (channel 2, green) for cells shown in (A). Fluorescence intensity analysis between the signal corresponding to rh-α-Gal-A and the signal corresponding to LysoTracker staining showing good correlation with a Pearson’s coefficient (R2) for the colocalization volume R2 = 0.62. (C) The table represents the measurement of areas of colocalization pixels vs. total cells area (image A, co-localization regions, yellow color). (D) Immunofluorescence images of nocodazole treated fibroblasts: fluorescence-labeled rh-α-Gal-A (left top panel), LysoTracker (left bottom panel), merge red and green images using ImageJ-win64 (right top panel) and image of co-localization areas (yellow) (right bottom panel). (E) 2D intensity histogram analysis between the signal corresponding to rh-α-Gal-A and the signal corresponding to LysoTracker showing low correlation with a Pearson’s coefficient (R2) for the colocalization volume R2 = 0.62. (F) The table represents the measurement of areas of colocolized pixels and all cells areas (image D, co-localization regions, yellow color); Table S1: Characteristics of cells used in this study and summary of a-Gal enzyme activity.
