# Supplementary material for: Rapid Clathrin-Mediated Uptake of Recombinant α-Gal-A to Lysosome Activates Autophagy
Source: Biomolecules. 2020 May 30;10(6):837. doi: 10.3390/biom10060837 (PMC7356514; doi:10.3390/biom10060837)
Supplement: Supplementary file 1 [file biomolecules-10-00837-s001.pdf]

biomolecules

MDPI

Supplemental Materials:

Table S1: Characteristics of cells used in this study and summary of a-Gal enzyme activity.

Cell Line	Origin	Disease Model	*GLA* variant	∝-Gal-A activity (nmol/mg/hr)
**HEK293**	Human embryonic kidney	control		220±31
**THP-1**	Monocytic cell derived from acute leukemia	control		155±24
**HUVEC**	Primary human umbilical vein endothelial cells	control		28.7±8
**Fibroblast-C**	Primary Dermal Fibroblast	control		49.6±23
**Fibroblast-FD-1**	Primary Dermal Fibroblast	FD	V269E	9.1±0.6
**Fibroblast-FD-2**	Primary Dermal Fibroblast	FD	Y134D	6,7±0.6
**UKEC-C**	Urine derived kidney epithelial cells	control		114±66
**UKEC-FD-1**	Urine derived kidney epithelial cells	FD	c.194+1/195-1 deletion	3.8±3.9
**UKEC-FD-2**	Urine derived kidney epithelial cells	FD	C223Y	6.0±0.64
**PBMC**	Peripheral Blood Mononuclear Cells	control		16.7±1.5

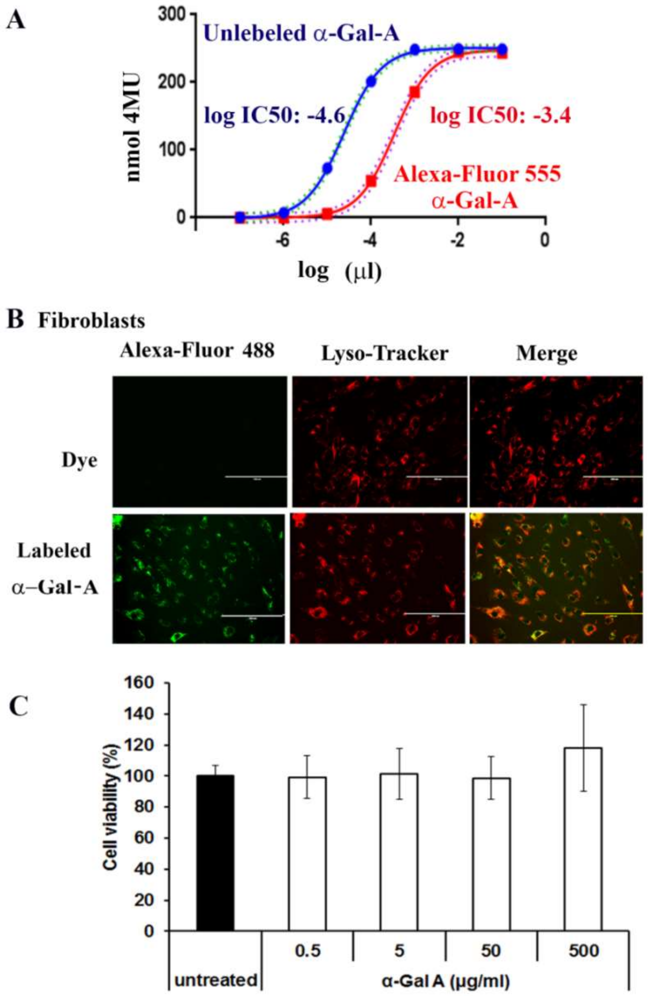

Figure S1. (**A**) The titration of rh-α-Gal-A enzyme activity pre- and post-labeling towards the artificial substrate 4-MUI. (**B**) Immunofluorescence images of fibroblasts treated with Alexa-Fluor 488-fluorescent dye alone and 488-fluorescence-labeled rh-α-Gal-A (green) for 1h and stained with LysoTracker (red). (**C**) Effects of rh-α-Gal-A on cell viability. HEK293 cells were treated with different concentrations of unlabeled rh-α-Gal-A fro 6h. The CCK-8 assay was performed to measure cell viability.

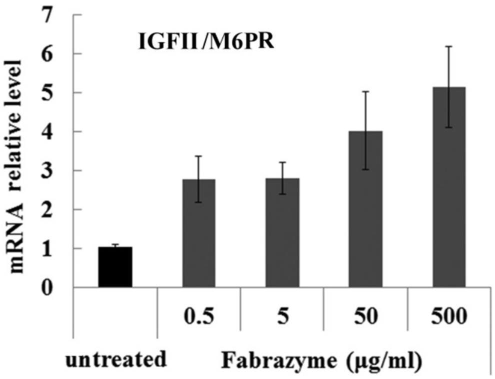

Figure S2. HEK293 cells were treated 6 h with the indicated concentrations of rh-α-Gal-A, or vehicle control (untreated), and real-time-PCR analysis of IGFII/M6PR was performed: 3 separate experiments.

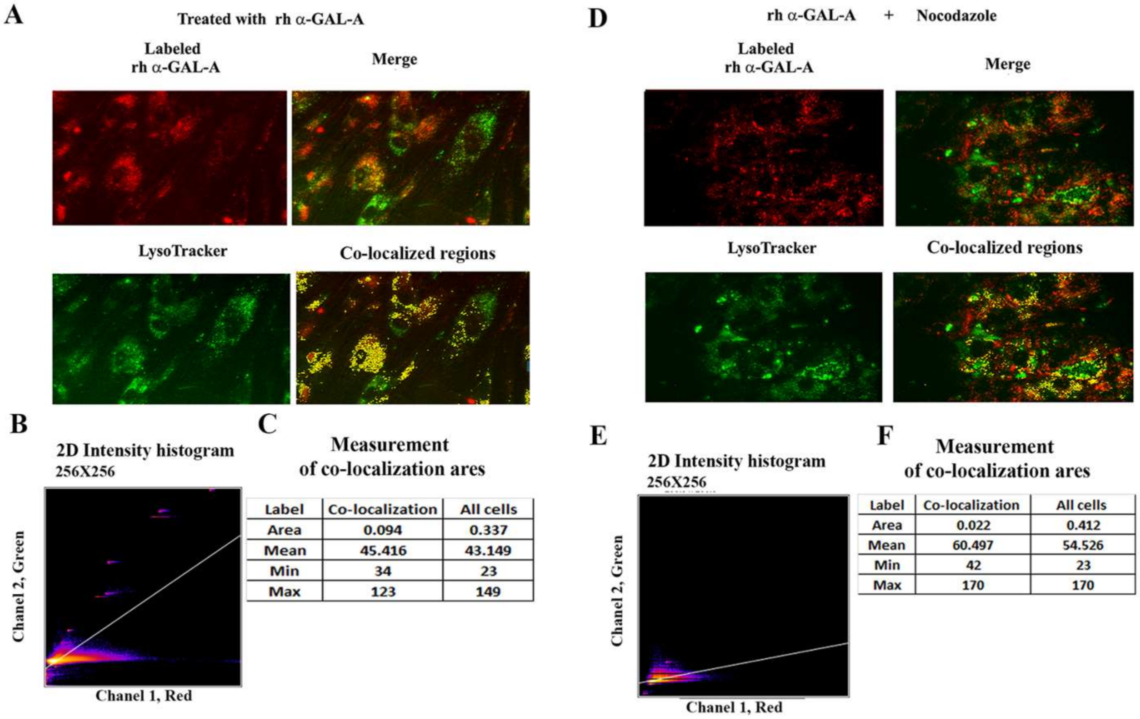

Label	Co-localization	All cells
Area	0.094	0.337
Mean	45.416	43.149
Min	34	23
Max	123	149

Label	Co-localization	All cells
Area	0.022	0.412
Mean	60.497	54.526
Min	42	23
Max	170	170

Figure S3. Colocalization analysis of immunofluorescence images (Figure 5B) of control fibroblasts treated with fluorescence-labeled rh-α-Gal-A (red) and stained with LysoTracker (green) with and without nocodazole treatment. (**A**). Immunofluorescence images of untreated fibroblasts: fluorescence-labeled rh-α-Gal-A (left top panel), LysoTracker (left bottom panel), merge red and green images using ImageJ-win64 (right top panel) and image of co-localization areas (yellow) (right bottom panel).(**B**). 2D intensity histogram of the red and green pixels in the images labeled rh-α-Gal-A (channel 1, red) and LysoTracker staining (channel 2, green) for cells shown in A. Fluorescence intensity analysis between the signal corresponding to rh-α-Gal-A and the signal corresponding to LysoTracker staining showing good correlation with a Pearson's coefficient (R^2) for the colocalization volume $R^2 = 0.62$.(**C**). The table represents the measurement of areas of colocalization pixels vs. total cells area (image A, co-localization regions, yellow color). (**D**). Immunofluorescence images of nocodazole treated fibroblasts: fluorescence-labeled rh-α-Gal-A (left top panel), LysoTracker (left bottom panel), merge red and green images using ImageJ-win64 (right top panel) and image of co-localization areas (yellow) (right bottom panel).(**E**). 2D intensity histogram anlysis between the signal corresponding to rh-α-Gal-A and the signal corresponding to LysoTracker showing low correlation with a Pearson's coefficient (R^2) for the colocalization volume $R^2 = 0.62$.(**F**). The table represents the measurement of areas of colocolized pixels and all cells areas (image D, co-localization regions, yellow color).
